# Oliguria in critically ill patients: a narrative review

**DOI:** 10.1007/s40620-018-0539-6

**Published:** 2018-10-08

**Authors:** Sebastian J. Klein, Georg F. Lehner, Lui G. Forni, Michael Joannidis

**Affiliations:** 1 Division of Intensive Care and Emergency Medicine, Department of Internal Medicine, Medical University Innsbruck, Anichstraße 35, 6020 Innsbruck, Austria; 2 Department of Clinical and Experimental Medicine, Faculty of Health Sciences, University of Surrey, Guildford, UK; 3 Intensive Care Unit, Royal Surrey County Hospital NHS Foundation Trust, Guildford, UK

**Keywords:** Oliguria, Acute kidney injury, Renal replacement therapy, Biomarker

## Abstract

Oliguria is often observed in critically ill patients. However, different thresholds in urine output (UO) have raised discussion as to the clinical importance of a transiently reduced UO of less than 0.5 ml/kg/h lasting for at least 6 h. While some studies have demonstrated that isolated oliguria without a concomitant increase in serum creatinine is associated with higher mortality rates, different underlying pathophysiological mechanisms suggest varied clinical importance of reduced UO, as some episodes of oliguria may be fully reversible. We aim to explore the clinical relevance of oliguria in critically ill patients and propose a clinical pathway for the diagnostic and therapeutic management of an oliguric, critically ill patient.

## Introduction

Oliguria is observed in many critically ill patients and was one of the very first “biomarkers” of acute kidney injury (AKI) [1] being described as early as 100–200 AD by Epheseus and Galen with Galen proposing a differential diagnostic pathway for the workup of an oliguric patient [2, 3]. The English physician Heberden later described renal failure accompanied by oliguria, at that time named as ‘ischuria renalis’ [1]. Although many new AKI biomarkers have been identified, oliguria is still of clinical importance, indeed all classification systems for AKI (RIFLE (Risk, Injury, Failure, Loss of kidney function, and End-stage kidney disease) [4], AKIN (Acute Kidney Injury Network) [5] and the KDIGO (Kidney Disease: Improving Global Outcomes) AKI criteria [6]) include urine output (UO) as part of the diagnostic criteria for AKI. Oliguria is most commonly defined as a urine output < 0.5 ml/kg over a period of 6 h although different time periods as well as cut-offs have been described varying between 1 and 24 h [6, 7].

## Epidemiology

A recent evaluation from intensive care patients found that nearly 50% experienced an episode of oliguria during their intensive care unit (ICU) stay [8]. Vaara et al. evaluated 2160 critically ill patients and found that nearly 30% of these patients experienced oliguria (UO < 0.5 ml/kg/h for ≥ 6 consecutive hours) and therefore fulfilled the criteria for oliguric AKI [9]. As was shown for an isolated serum creatinine (sCr) increase, oliguria per se is also associated with increased mortality. For example, one study found an increased ICU mortality in oliguric patients without a change in sCr (8.8%), which was similar to an isolated increase in sCr (10.4%). In both cases mortality rates were significantly higher than in patients without AKI (1.3%) [8]. A further large retrospective study with over 23,000 patients documented more adverse events in patients who reached maximum AKI stage according to both UO and creatinine criteria compared to UO or creatinine criteria alone. The observed mortality rates in patients with AKI at 90-days and 1-year when classified either by UO (19.1% and 28%) or creatinine (22.9% and 31.9%) were similar, but were much higher at 90 days and one year, respectively, in patients who reached maximum AKI stage using both criteria (37.8% and 47.9%) [10]. Similar findings have been reported previously by analyzing the multicenter international SAPS 3 database with data on 14,000 patients [11, 12]. Furthermore, in a recently published trial, a urine output < 0.5 ml/kg/h was associated with lower rates of resolving AKI (HR 0.31; 95% CI 0.20–0.47) [13]. This was also found in another trial of 264 patients receiving CRRT for AKI after cardiac surgery, where significantly fewer patients with oliguria recovered renal function (40.2% vs. 62.5%, p < 0.001) [14].

However, despite a seemingly large body of evidence, it must be emphasised that most of these studies were retrospective with heterogeneous patient groups. Another limitation of many studies examining oliguria and UO thresholds is the fact, that, with exception of two studies [15, 16], volume status was not specifically reported before assessment of oliguria [8, 9]. This might be of importance, since correction for volume expansion improves prediction of both UO and sCr KDIGO criteria with respect to mortality [17]. Furthermore, only one study adjusted for diuretic use in multivariate analysis [9], whilst most studies did not emphasize this important point [7, 12, 13]. Moreover, different time intervals for UO and sCr measurement were used and that the definition of baseline creatinine differed across studies. These factors can all contribute to a variable reported prevalence of AKI.

The suggested consequences of these results is that, oliguria without a concomitant elevation of SCr may be of clinical importance, as it is still associated with increased mortality in ICU patients. However, interpretation of isolated oliguria as with many observations in medicine must be taken in context.

## Pathophysiology

**Fig. 1:**
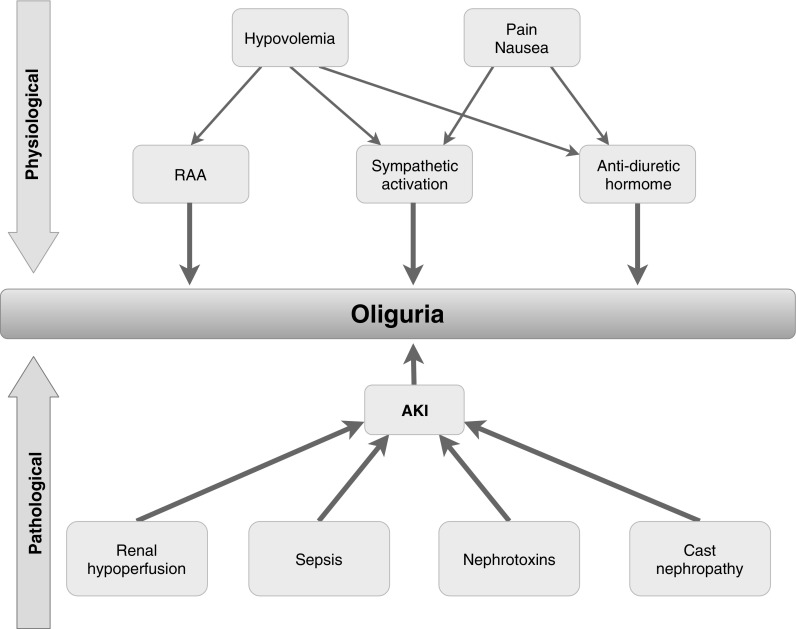
Physiological and pathological stimuli leading to oliguria (*RAA* renin–angiotensin–aldosterone system, *AKI* acute kidney injury)

When considering the etiology of oliguria, one has to distinguish whether it is a normal physiological response or reflects an underlying pathological process (Fig. 1). Physiological oliguria may result, for example, from antidiuresis due to hypovolemia, after significant food and water fasting and also after ultra-endurance events, where oliguria is experienced by some athletes during and up to some hours after the event [18], however, these episodes of oliguria seem to be fully reversible, and do not increase the risk for subsequent kidney injury [19]. Transitory oliguria is also frequently observed in post-operative non-critically ill patients associated with vasopressin-release and activation from the sympathetic nervous system from pain or nausea [20].

In critically ill patients, however, different pathophysiological pathways may lead to oliguria. First, there is the neuro-hormonal pathway influencing kidney function through activation of the sympathetic nervous system leading to elevated activity of the renin-angiotensin-aldosterone system (RAAS), higher levels of circulating vasopressin and activation of the tubuloglomerular feedback system (TGF). This results in systemic vasoconstriction, reduced renal filtration as well as water and sodium retention [21]. In patients suffering from chronic heart failure, those neuro-humoral effects can often be observed, furthermore these neuro-humoral response are responsible for certain types of AKI like the hepatorenal syndrome (HRS) [22, 23].

Absolute (hypovolemia) and relative (hemodynamic perturbations) reductions in effective blood volume may lead to a reduced renal blood flow (RBF). However, this reduction in RBF alone is not usually sufficient to lead to a reduced glomerular filtration rate (GFR). Furthermore, in some situations, like sepsis-associated AKI, RBF may be preserved or even be increased [24]. What seems to be more influential than global RBF is intra-renal blood flow influenced by pre- and post-glomerular resistance and intra-renal shunting. This dissociation between global and intra-renal blood flow is supported by a study measuring the interlobar artery resistivity index, which found that in response to fluid administration, even without a relevant (> 10%) change in MAP and therefore in global RBF, an improvement in intra-renal perfusion translating into an increased UO was observed [25].

While pre-renal reasons for oliguria, like activation of the RAAS are thought to be—at least in part—rapidly reversible, this often does not apply to oliguria/AKI resulting from direct pathological insults to the kidney. Here, oliguria is the consequence of diminished GFR, tubular obstruction from tubular casts and back-leak of tubular solutes. Inflammation and sepsis is another important mechanism for oliguria as demonstrated by the fact, that sepsis is often accompanied by oliguria [26]. While systemic vasodilation in sepsis is predominant, macro- and microcirculatory alterations may diminish blood flow to certain regions of the kidney. This leads to the phenomenon, that despite an increase in renal blood flow (RBF), oliguria followed by AKI may rapidly develop. Furthermore, besides circulatory changes, immunologic and inflammatory mechanisms (damage- and pathogen-associated molecular pattern molecules [DAMPs, PAMPs], Microvesicles and TNF-α) [24] may lead to endothelial injury, among others. This endothelial injury may induce increased vascular permeability, followed by interstitial edema [27–30].

## Urine output threshold

As outlined, consensus opinion defines oliguria as a urine output of < 0.5 ml/kg/h for more than 6 h. This threshold is predictive for AKI defined by increase in SCr in critically ill patients but recent findings in surgical patients question this threshold for the perioperative period. Mizota et al. found that a cut-off < 0.3 ml/kg/h was independently associated with postoperative AKI (adjusted OR 2.65; 95% CI 1.77–3.97) in a Japanese patient cohort. No correlation was found for a cut-off between 0.3 and 0.5 mL/kg/h and the development of postoperative AKI [16]. These findings provide further support for interpreting oliguria within the clinical context in that a relatively reduced urine output albeit greater than 0.3/ml/kg/h is likely to represent a (reversible) physiological response to perioperative stimuli including intravascular hypovolemia, reduced renal perfusion due to hypotension and release of anti-diuretic hormone in response to nausea or pain [31]. Ralib et al. were able to confirm by analyzing 725 admissions to a general ICU, that a urine output threshold of 0.5 ml/kg/h may be too liberal given that a threshold for 6-h UO of 0.3 ml/kg/h was best associated with the combined endpoint of dialysis or mortality. Interestingly, they found that the optimal threshold of UO was linearly related to the duration of the collection period [32], which might reflect the presence of non-pathological mechanisms that occur in addition to major pathophysiological processes within the observed time period. This was supported by Prowle et al. who showed significant improvement of prediction of worsening AKI after an episode of at least 12 h of oliguria [33]. This may explain why the optimal UO threshold used to identify relevant end-point-determining pathophysiologic events increases along with the duration of collection. Another important fact when considering weight-based urine output criteria is that in obese patients, these may lead to an overdiagnosis of AKI and therefore the ideal body weight should be used in calculating the urine output rather than the actual body weight [34, 35].

## Diagnostic and therapeutic approach

When considering the diagnostic and therapeutic options in treating an oliguric patient a step wise approach may be employed. Firstly, hemodynamic stabilization must be achieved taking into consideration premorbid parameters if known. Subsequently, the patient’s response to diuretics in the standardized form of the Furosemide Stress Test (FST) may be tested, after euvolaemia has been established [36]. Novel biomarkers like neutrophil gelatinase-associated lipocalin (NGAL) may also improve risk stratification although there is still lack of clear evidence to strongly support the routine use of such biomarkers in isolated oliguria [37, 38]. If the patient’s UO does not improve after hemodynamic stabilization and is unresponsive to diuretics [39], further AKI workup should be conducted [6]. Finally, while an adequate volume status should be achieved, volume overload must be avoided and possibly treated, either with diuretics where responsive or ultimately with renal replacement therapy (RRT) [39].

### Hemodynamic stabilization (Step 1)

**Fig. 2:**
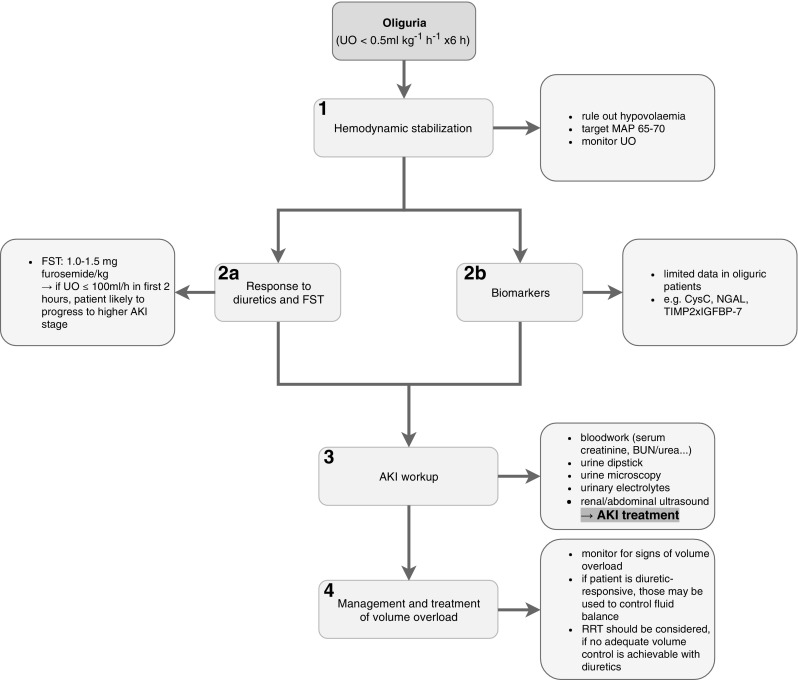
4-step approach (step 1 - hemodynamic stabilization, step 2a - response to diuretics and FST, step 2b - biomarkers [steps 2a and 2b may be considered as alternative approaches], step 3 - AKI workup, step 4 - management and treatment of volume overload)  to the clinical management of the oliguric patient (*UO* urine output, *MAP* mean arterial pressure, *FST* furosemide stress test, *AKI* acute kidney injury, *CysC* cystatin C, *NGAL* neutrophil gelatinase-associated lipocalin, *TIMP-2 x IGFBP-7* tissue inhibitor of metalloproteinase 2 × insulin-like growth factor binding protein 7, *BUN* blood urea nitrogen, *RRT* renal replacement therapy)

Initially, hypovolemia must be excluded in an oliguric patient and corrected to obtain adequate renal perfusion although care should be taken to avoid volume overload [39]. Starches should be avoided as they may lead to osmotic tubular damage. If large fluid volumes are needed for fluid resuscitation, balanced crystalloids are preferable. An adequate hemodynamic state should be achieved to ensure proper RBF. Ideally, vasopressor therapy should target a MAP of 65–70 mmHg, unless the patients suffers from chronic hypertension (Fig. 2) [39].

When a patient is developing oliguria, UO should be monitored. This is easily achievable in ICU patients, who often are catheterized and hourly urine monitoring is therefore feasible, but should also be achieved on general wards. A retrospective cohort study including over 15,000 adult ICU patients found that intensive monitoring of UO yields significantly higher rates of AKI (OR 1.22) and was even associated with improved survival, but only among patients who suffered from AKI [40].

### Response to diuretics and furosemide stress test (Step 2a)

If the patient remains oliguric after achieving adequate volume and hemodynamic status, the response to diuretics may be tested. While diuretics are already frequently used in oliguric patients [41], it is advisable to utilize a standardized approach to maximize their diagnostic ability.

A common difficulty when managing patients either at risk of, or with, AKI is to predict which patients will progress to a higher stage/severity of illness. A tool that may be used for risk stratification is the furosemide stress test (FST), a standardized test of the functional integrity of the tubule and which was developed to aid such decision making. A urinary output of ≤ 100 ml/h in the first 2 h following a dose of 1.0–1.5 mg furosemide/kg (FST non-responsive) predicted progression to a higher AKI stage with both high sensitivity and specificity [36]. Clearly, when conducting a FST it is important that the patient is not hypovolemic with blood pressure and heart rate closely monitored. The FST may also turn out to be a useful tool for decision support on initiating renal replacement therapy (RRT). In a recent feasibility trial, only 13.6% of FST responsive patients ultimately needed RRT, while of patients who were FST non-responsive 75% required RRT [42]. This is a novel aspect because oliguria per se does not reliably predict the requirement of RRT in critically ill patients [43]. If a patient is responding to diuretics, these should be used for volume control [39, 44].

### Biomarkers (Step 2b)

Biomarkers may be used to assess the risk of AKI as an underlying reason for oliguria and to guide appropriate therapeutic actions. To date, one randomized trial has evaluated the implementation of a care bundle in high risk patients after cardiac surgery, who had elevated levels of TIMP-2 × IGFBP-7. This approach lead to a reduced rate of AKI as mainly diagnosed by UO criteria [45] suggesting a possible benefit of biomarkers in risk assessment.

However, few studies have assessed biomarkers for predicting worsening AKI specifically in oliguric patients [37, 38, 46]. One trial, measuring biomarkers in blood (neutrophil gelatinase-associated lipocalin [NGAL] among others) and urine of oliguric patients, tried to utilize these biomarkers for better risk stratification of poor renal outcome. However, those biomarkers were not better than sCr leading to the conclusion that not all episodes of oliguria carry the same risk for adverse outcomes, which could have diminished the predictive ability of those biomarkers [38]. In the study by Egal et al., neutrophil gelatinase-associated lipocalin (NGAL) was able to discriminate between patients experiencing functional oliguria and those who developed AKI according to SCr criteria [37]. Another study, which evaluated the prediction of fluid responsiveness of uNa^+^, fractional excretion of sodium (FE_Na_) and the fractional excretion of urea in oliguric patients, found that those biomarkers had no significant predictive value [46]. While NGAL was able to help in differentiating reversible from non-reversible forms of oliguria in the study by Egal et al. [37], Legrand et al. reported no better risk stratification by utilizing NGAL as compared to SCr [38]. In light of these results, further studies are necessary, before novel biomarkers are routinely employed for risk stratification in oliguric patients [11].

When evaluating biomarker levels in urine, controversy exists, particularly as to whether those biomarker levels should be normalized to urinary creatinine, thereby accounting for urine volume. In a recent systematic review, which analyzed the predictive ability of biomarkers regarding the necessity of RRT, there was a slight trend towards biomarkers normalized to creatinine performing better than non-normalized. However, these differences were not statistically significant [43].

### AKI workup (Step 3)

A baseline workup should follow, including bloodwork (sCr, BUN or urea, serum electrolytes etc.), urine dipstick analysis, urine microscopy (urinary sediment), urinary electrolytes and renal/abdominal ultrasound.

A more specific diagnostic workup may follow, depending on the context, severity, duration and local availability which may include the assessment of an autoimmune profile (among others: anti-nuclear antibody [ANA], anti-neutrophil cytoplasmic antibody [ANCA], anti-glomerular basement membrane antibody [anti-GBM]), a renal biopsy and additional laboratory tests (e.g., in case of suspected rhabdomyolysis: serum creatinine kinase and myoglobin; in case of suspected cardio-renal syndrome: N-terminal pro-brain natriuretic peptide [NT-proBNP], etc.) [34]. Reversible (exogenous) causes for oliguria should be excluded. For example, when assessing a catheterized patient, catheter dysfunction should be excluded and bedside ultrasound can easily rule out postrenal or obstructive causes of oliguria. Ultrasound can also be helpful in assessing renal perfusion at the bedside through the Doppler-based renal resistive index (RI) [47].

Treatment of a patient with oliguria should primarily rely on the guidelines for AKI treatment [6, 39, 44, 48]. For patients at high-risk of AKI, nephrotoxic agents should be discontinued where possible, volume status and (renal) perfusion pressure should be maintained and monitored, possibly by utilizing invasive monitoring [39]. A more detailed overview of the diagnostic workup and therapeutic options of AKI is outside of the focus of this paper and can be found elsewhere [34, 39].

### Management and treatment of volume overload (Step 4)

If deteriorating renal function and subsequently diminishing urine output are used as a trigger for treatment, including fluid loading, the reduced urine output may, as a consequence, contribute to fluid overload, which may in itself lead to worsening AKI where the oliguria does not respond to these measures. Interestingly, a retrospective analysis of the Fluid and Catheter Treatment Trial, found that when a fluid bolus was given for shock or oliguria (< 0.5 mL/kg/h), no significant changes in MAP, heart rate, CVP, pulmonary artery occlusion pressure or UO were observed 1–4 h after the bolus [15].Therefore, relying on UO as a trigger for fluid administration may lead to an overestimation of the achievable effect. Especially in the setting of oliguria, one has to be aware, that inconsiderate fluid loading may lead to volume overload, which is associated with increased mortality [39, 49, 50].

Possible signs of fluid overload may include peripheral edema [51] and an increase in the central venous pressure (CVP) as sonographically determined via the diameter of the vena cava inferior [51, 52]. If invasive monitoring (e.g., Pulse Contour Cardiac Output—PiCCO) is used, the global end-diastolic volume index (GEDI), extravascular lung water (EVLW) and stroke volume variation (SVV) may be also utilized to identify possible fluid overload. In patients that are diuretic-responsive, diuretics may be used to control fluid balance and to avoid volume overload [30]. Of note, a feasibility study in septic patients, applying a fluid restrictive regimen has shown a decreased rate of AKI [45].

If adequate volume control is not achievable with diuretics, RRT should be considered [39, 44]. In fact, oliguria was the leading reason for commencement of RRT, both in the RENAL (approx. 60%) [53] and the AKIKI trial (38%) [54].

## Conclusion

To date, oliguria remains an important biomarker for renal function as well as volume status. However, care must be taken to interpret diminished UO in a broader clinical context, as different pathophysiological mechanisms may lead to oliguria. Our proposed 4-step approach to the management of an oliguric patient may aid in clinical decision making.
